# Practice and knowledge of dietary supplement consumption among Indonesian adults post-delta wave of the COVID-19 pandemic

**DOI:** 10.12688/f1000research.129045.2

**Published:** 2023-05-04

**Authors:** Annette d'Arqom, Mhd Zamal Nasution, Sharifah Zamiah Syed Abdul Kadir, Junaidah Yusof, Kayatri Govindaraju

**Affiliations:** 1 Translational Medicine and Therapeutics Research Group, Universitas Airlangga, Surabaya, 60131, Indonesia; 2 Department of Anatomy, Histology, and Pharmacology, Faculty of Medicine, Universitas Airlangga, Surabaya, East Java, 60131, Indonesia; 3 Postgraduate School, Universitas Airlangga, Surabaya, East Java, 60286, Indonesia; 4 Department of Pharmacology, Faculty of Medicine, University of Malaya, Kuala Lumpur, 50603, Malaysia; 5 School of Human Resource Development & Psychology, Faculty of Social Sciences & Humanities, Universiti Teknologi Malaysia, Johor Bahru, 81310, Malaysia; 6 Faculty of Pharmacy, University of Malaya, Kuala Lumpur, 50603, Malaysia

**Keywords:** awareness, COVID-19, diet, healthy lifestyle, herbal, mineral, vitamin, sociodemographic

## Abstract

**Background:** Increasing dietary supplement (DS) consumption was observed during the COVID-19 pandemic, including during the post-Delta wave period. This study aimed to measure the practice of DS consumption and respondents’ knowledge of DS.

**Methods:** An internet-based survey was distributed from October-December 2021 and obtained 541 valid and completed responses. Descriptive analysis was performed to present the practice of DS consumption, including frequency, duration, aim, preferable dosage form etc. Level of knowledge on DS principles, side effects and regulation were also measured. Inferential analyses were conducted to determine the predictors of the respondents’ DS practice and level of knowledge.

**Results:** Data from 541 valid responses showed that 77.63% of respondents consumed DS in the last 3 months, with only 59.52% reporting also consuming DS before the COVID-19 pandemic. One half of the respondents had good knowledge about DS; however, some knowledge regarding side effects and possible drug-supplement interaction needed improvement. Their DS consumption practice was affected by their economic status and history of contracting COVID-19. Nevertheless, the level of knowledge was not affected by the sociodemographic factors and DS supplement experience.

**Conclusions:** Taken together, the practice of self-consumption of DS in Indonesia is increasing; hence, knowledge of DS is necessary to avoid detrimental effects that might occur in the future. Increasing access to information on better labelling and educating consumers about DS are important actions to consider.

## Introduction

United States Food and Drugs Administration (USFDA) defined dietary supplements (DS) as substances containing vitamins, minerals, amino acids or enzymes, or herb/botanical products that complete the diet's nutritional value. However, DS is not intended for treating, curing, preventing, or diagnosing diseases ( FDA, 2022). Despite that definition by the USFDA, much evidence-based research, from fundamental animal studies to randomized clinical trials has shown the effectiveness of DS consumption in preventing disease, improving health, and reducing disease symptoms ( Kaviani *et al.*, 2020; Shinto *et al.*, 2014; Sim *et al.*, 2022). Moreover, the global consumption of DS has been steadily increasing for the last decade ( Kantor *et al.*, 2016; Kelly *et al.*, 2005). Since 2020, the public's interest in DS use has increased in the wake of COVID-19 worldwide ( d'Arqom *et al.*, 2021; Hamulka *et al.*, 2021; Lordan, 2021; Speakman *et al.*, 2021). At the beginning of the pandemic, some DS recorded higher demand and consumption rates due to the perception that DS ingredients might boost immune function and reduce inflammation to help prevent COVID-19 ( d'Arqom *et al.*, 2021; Hamulka *et al.*, 2021; Mohsen *et al.*, 2021).

The COVID-19 outbreak has claimed a total of 6,540,487 lives as of October 2022 ( WHO, 2022). More than 90% of COVID-19 deaths involved a pre-existing medical condition, of which 66% of the patients had metabolic syndrome, a pathologic condition due to obesity, insulin resistance, hypertension and hyperlipidemia ( Li *et al.*, 2021). Patients who present with one or two comorbidities are at a higher risk of low survival following COVID-19 transmission. This is due to impaired function resulting from the secondary effects of HDL cholesterol, as observed by Kitchens *et al.* (2003). Moreover, the decline in respiratory functions is likely caused by prolonged systemic inflammation, as indicated by high c-reactive protein levels ( Chen *et al.*, 2014; Kim *et al.*, 2017). Additionally, the high risk of cardiac arrest is attributed to the underlying comorbidities of metabolic syndrome, as highlighted by Hess *et al.* (2017).

All the mentioned medical complications could be prevented by early detection, being active, and meeting the physiological demands of essential vitamins and minerals. It has been established that vitamin C, B6, D, and E deficiency were associated with an increased risk of metabolic syndrome ( Beveridge *et al.*, 2015; Godala *et al.*, 2017; Kodentsova *et al.*, 2019). In light of the pandemic, there has been a significant shift in the way healthcare providers and the general public perceive dietary supplements. For example, there has been an increased recognition of their potential to mitigate the severity of COVID-19 infections. Notably, countries such as Indonesia have been increasingly providing essential dietary supplements to high-risk populations to reduce the risk of hospitalization and adverse post-complications of COVID-19 transmission ( Kemenkes, 2022; Weir *et al.*, 2020). A study conducted in the UK on 445,850 participants showed a significant association between the use of multivitamins, omega-3 fatty acids and vitamin D _3_ supplements with a lower risk for SARS-CoV-2 ( Louca *et al.*, 2021). However, uncontrolled and overconsumption of DS might lead to unwanted effects, such as kidney problems, cancer and drug-herbs interactions ( Agbabiaka *et al.*, 2018; Asher *et al.*, 2017; d'Arqom *et al.*, 2020; Peng *et al.*, 2004).

In Indonesia, although the use of DS is high, little published data is available regarding public practice and knowledge towards DS and their safety. As such, more research on public awareness of DS is essential to gain a better understanding of people's beliefs and expectations regarding DS. Therefore, this study aimed to investigate the practice and knowledge of the general public in Indonesia regarding DS during the post-Delta wave of the COVID-19 pandemic. In addition, the predictive factors of DS consumption and level of knowledge were further analyzed.

## Methods

### Study design

This research employed a cross-sectional design. The primary data were collected using an internet-based questionnaire ( www.surveyplanet.com). The questionnaire was distributed to the adult population in Indonesia through email and social media using convenience sampling methods from October to December 2021, and multiple responds from single device were prevented based on respondents’ IP address. This study followed the Checklist for Reporting Results of Internet E-Surveys (CHERRIES) and The Strengthening the Reporting of Observational Studies in Epidemiology (STROBE) guidelines ( Eysenbach, 2004; Vandenbroucke *et al.*, 2007). The ethics clearance was issued by the Faculty of Medicine, Universitas Airlangga No. 244/EC/KEPK/FKUA/2021.

### Participants

The inclusion criteria were Indonesian adults older than 18 years old and residing in Indonesia during the post-Delta wave of the COVID-19 pandemic. Sample size was calculated using sample size calculator ( www.raosoft.com) with 5% margin of error, 95% confidence level, and population number filled with 100,000, resulting in 383 minimum respondents. Respondents were recruited through announcements distributed in social media and email using non-probability sampling.

### Data collection

Respondents independently filled in the online questionnaire using their own device, and the estimated survey length was 12 minutes. To measure respondents' practice and awareness on DS, a set of questionnaires containing three sections was distributed online. The sections comprised basic demographic information, supplement consumption practice, and DS knowledge. The first section consisted of basic demographic factors such as age, sex, domicile, education, occupation, field of study/work, COVID-19 infection status, comorbidity, marital status, and self-claimed economic status. The second section consisted of 17 questions regarding their supplement consumption practice, which included respondents’ consumption of DS, duration, frequency, numbers, place to buy, aims, benefit, and source of information. And the last section comprised 16 questions to measure DS knowledge, including their knowledge on the regulation, side effects, and benefit. The questionnaire was modified from the Prevalence and Awareness Concerning Dietary Supplement Use among Saudi Adolescents ( Alfawaz *et al.*, 2020) and Knowledge about dietary supplements and trust in advertising them: Development and validation of the questionnaires and preliminary results of the association between the constructs ( Karbownik *et al.*, 2019). The questionnaire was combined and validated by two pharmacologists, a pharmacist, and a psychologist, and was further tested on 20 respondents to ensure that the content and terms used in the questionnaire were relevant and understandable. The reliability measurement on the survey data showed a Cronbach alpha coefficient of 0.750 for DS knowledge. As the reliability value for the preliminary pilot testing is at an acceptable level, the real data collection for this study was proceed. A copy of the survey instrument can be found under *Extended data* ( d’Arqom, 2022).

### Ethical considerations

This study followed the Helsinki declaration and approved by the Health Research Ethics Committee, Faculty of Medicine, Universitas Airlangga (No. 86/EC/KEPK/FKUA/2021). The aims of the study, the consent, and the permit to publish their responses anonymously were provided in the landing page, prior completing the questionnaire. The respondents provided their consent by clicking the YES button before starting the questionnaire.

### Analytical procedures

Only the complete responses were processed and analysed. Respondents were grouped based on their sociodemographic factors. Supplement practice and awareness were measured using nominal or ordinal scales. Data were processed and analyzed using Microsoft Excel and SPSS 24.0 (IBM, Chicago, IL, USA). Graphs were visualized using GraphPad Prism 5.0. Descriptive statistical analyses were performed, including the frequency for each categorical variable. The answers to the knowledge survey were measured using correct/wrong/does not know options measured as 1/0/0. The total score was the summation of the 16 question scores and was grouped in four categories based on the total score of each respondent: poor (0-4), moderate (5-8), good (9-12) and excellent (13-16). The Chi-square test and Fisher's Exact test were performed to measure the difference between DS consumption and knowledge on each group. To investigate the predictors of DS consumption, binary logistic regression with 95% confidence intervals (95% CIs) was calculated. Moreover, to investigate the predictors of DS knowledge, ordinal logistic regression with 95% CI was performed. Prior to multivariate regression, univariate regression was performed to determine each sociodemographic factor that might affect DS consumption or level knowledge. Sociodemographic factors with p<0.25 were included to further multivariate regression using the backward elimination method ( Bursac *et al.*, 2008). Variables with p<0.05 from the multivariate regression analysis were considered significant predictive factors.

## Results

### Characteristics of respondents

**Figure 1.**
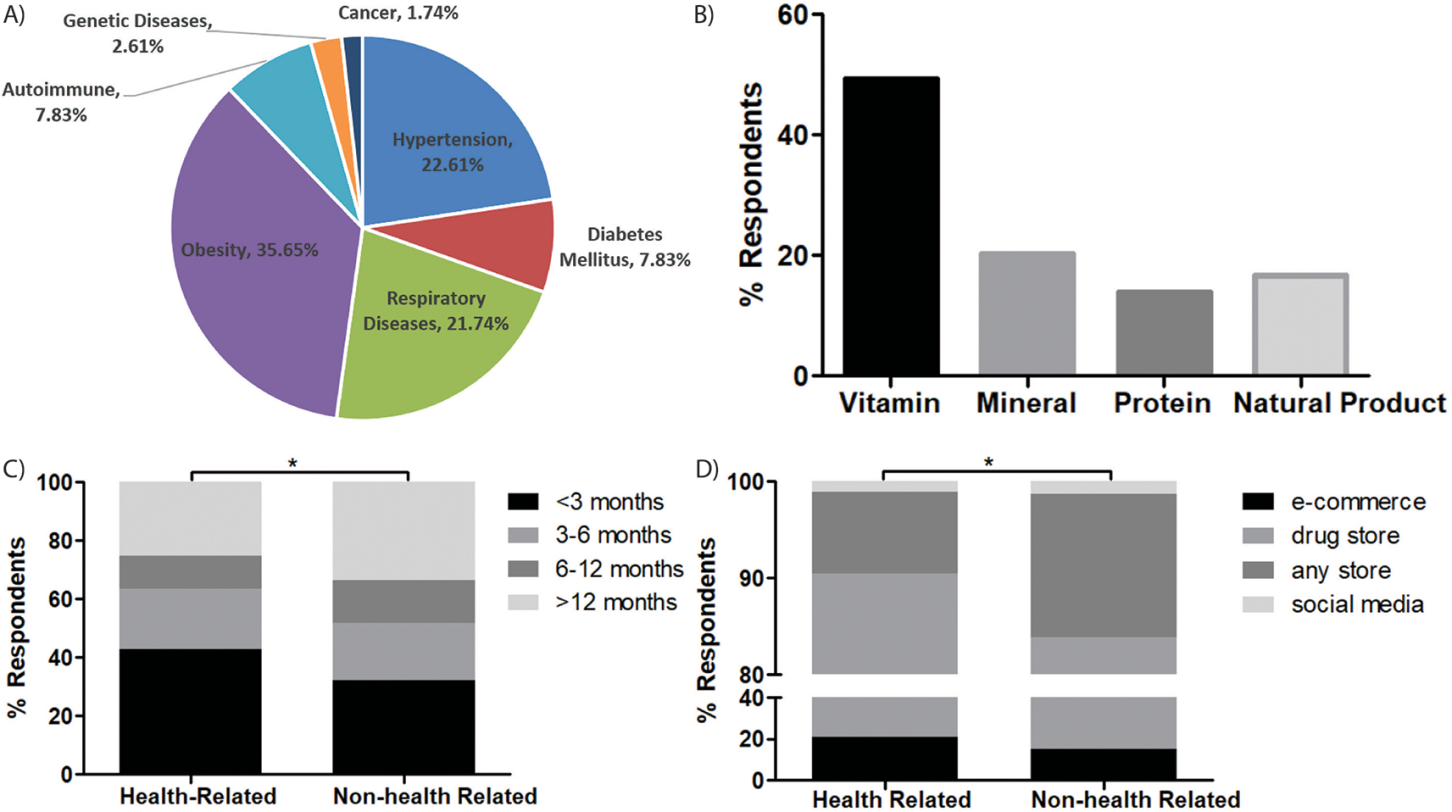
Comorbidity and DS consumption practice. (A) types of comorbidities of the respondents, (B) types of DS consumed by the respondents, (C) duration of DS consumption (D) Source of DS, * *p-value<0.05.*

Five hundred forty-eight respondents visited the informed consent page, and 541 completed and valid questionnaires used in the final analysis (completion rate was 98.72%). Around two thirds of respondents comprised young adults, females and unmarried individuals. The majority came from the main island, which is the most developed region in Indonesia (82.26%), with self-reported economic status, were average economic status (73.54%) and never had a COVID-19 positive status (78.19%). Table 1 summarizes the respondents’ sociodemographic characteristics. Hundreds (18.48%) were reported to have comorbidities such as obesity (35.65%), hypertension (22.61%), respiratory diseases (21.74%) and others (diabetes mellitus, autoimmune diseases, genetic diseases and cancer) ( Figure 1A). The full dataset can be found under *Underlying data* ( d’Arqom, 2022).

**Table 1.** Sociodemographic characteristic of respondents.

Sociodemographic factors	Total respondents (N=541)	Supplement consumer	Non-supplement consumer	X ^2^	*p*
**Age**				14.686	***0.005***
18-25	338 (62.48%)	245 (72.49%)	93 (27.51%)		
26-35	77 (14.23%)	65 (84.42%)	12 (15.58%)		
36-45	61 (11.28%)	54 (88.52%)	7 (11.48%)		
46-55	51 (9.43%)	45 (88.24%)	6 (11.76%)		
>55%	14 (2.59%)	11 (78.57%)	3 (21.43%)		
**Sex**				0.006	*1*
Male	213 (39.37%)	165 (77.46%)	48 (22.54%)		
Female	328 (60.63%)	255 (77.74%)	73 (22.26%)		
**Location**				0.466	*0.501*
Main Island	445 (82.26%)	348 (78.20%)	97 (21.80%)		
Outside Main Island	96 (17.74%)	72 (75%)	24 (25%)		
**Education**				11.203	***0.004***
High School graduate	240 (44.36%)	171 (71.25%)	69 (28.75%)		
Diploma or Undergraduate	217 (40.11%)	176 (81.11%)	41 (18.89%)		
Post graduate	84 (15.53%)	73 (86.90%)	11 (13.10%)		
**Work**				12.712	*0*
Employed	190 (35.12%)	164 (86.32%)	26 (13.68%)		
Unemployed	351 (64.88%)	256 (72.93%)	95 (27.07%)		
**Field of work/study**				2.783	*0.110*
Health-related	339 (62.66%)	271 (79.94%)	68 (20.06%)		
Non-health-related	202 (37.34%)	149 (73.76%)	53 (26.24%)		
**Confirmed COVID-19**				6.741	***0.009***
Never	423 (78.19%)	318 (75.18%)	105 (24.82%)		
Ever	118 (21.81%)	102 (86.44%)	16 (13.56%)		
**Marital status**				12.703	***0.000***
Married	165 (30.11%)	144 (87.27%)	21 (12.73%)		
Unmarried	376 (68.61%)	276 (73.40%)	100 (26.60%)		
**Economy status**				8.971	***0.011***
Below Average	29 (5.29%)	16 (55.17%)	13 (44.83%)		
Average	403 (73.54%)	314 (77.92%)	89 (22.08%)		
Upper Average	109 (19.89%)	90 (82.57%)	19 (17.43%)		

Notes: Boldface p-values indicate significant differences between groups using Chi-square or Fisher-Exact test.

### Practice of dietary supplement consumption

Our study found that 420 (77.63%) respondents consumed DS during the post-Delta wave of the COVID-19 pandemic. Forty percent reported they had never consumed DS before the pandemic. Several sociodemographic factors were associated with DS consumption, for example, middle-aged adults were more likely to consume DS compared with elderly and young adults ( *p=0.005*). Higher education, higher self-reported economic status, married status, those diagnosed with COVID-19 and working respondents were also more likely to consume DS ( *p=0.004, 0.011, 0.000, 0.009, 0.000*, respectively). No statistically significant differences were found in DS consumption among sex, domicile and field of work/study. The frequency of supplement consumption based on their sociodemographic factors is summarized in Table 1.

Almost one half of respondents consumed vitamins (49.28%), minerals (20.19%), natural products (16.71%) and protein or amino acids (13.82%) ( Figure 1B). Their aims of DS consumption were mostly for increasing immunity (81.19%), preventing disease (10.71%), and improving physical appearance (8.10%). Almost 40% of the respondents had consumed DS for less than 3 months, while more than one quarter (28.33%) had consumed DS for more than one year. A different pattern of DS consumption emerged between respondents with health-related backgrounds and non-health-related backgrounds, as most of the last group took DS with longer duration than the first group ( *p=0.017*, Figure 1C).

The most preferred dosage form was capsule (49.76%), tablet (38.57%), liquid (7.62%) and powder (4.05%). Two thirds of the respondents consumed a single type of DS at one time, two types of DS (23.10%) and only 7.62% consumed three or more types of DS simultaneously. More than 40% of respondents consumed 2 to 5 times/weekly (43.10%), less than twice/weekly (33.33%), and 23.57% consumed it more than 5 times/weekly. Two thirds bought DS from drugs stores (69.29%), e-commerce (18.81%) and any store that sold DS (10.71%), while only 1.19% bought DS from social media. Even though the majority of respondents in both groups were more likely to buy DS from drug stores, the health-related groups were more likely to choose e-commerce as the second option, while the second option for non-health-related respondents was any store that sold DS and e-commerce platforms ( *p=0.025*, Figure 1D).

The primary sources of information were family members or friends (49.29%), and health professionals (32.86%), and the rest received information from academic journals, websites and TV/magazine advertisements. Surprisingly, 48.57% consumed DS for more than 3 months without healthcare consultation. Moreover, one half of respondents did not have an exact schedule for consuming DS, while only 30.71% consumed them on a scheduled basis. As expected, more than three quarters of respondents had missed consuming DS (82.62%). Almost three quarters (73.57%) felt the benefit of consuming DS. However, only one third would continue to consume DS if they faced financial difficulty (34.29%). More than one half of respondents consuming DS knew about their side effects and they consumed and endeavored to find information regarding suitable DS (67.38%). Differences in DS consumption between the two groups are summarized in S2 Table. A supplementary material can be found under Extended data ( d'Arqom, 2022).

### Knowledge on dietary supplements

**Figure 2.**
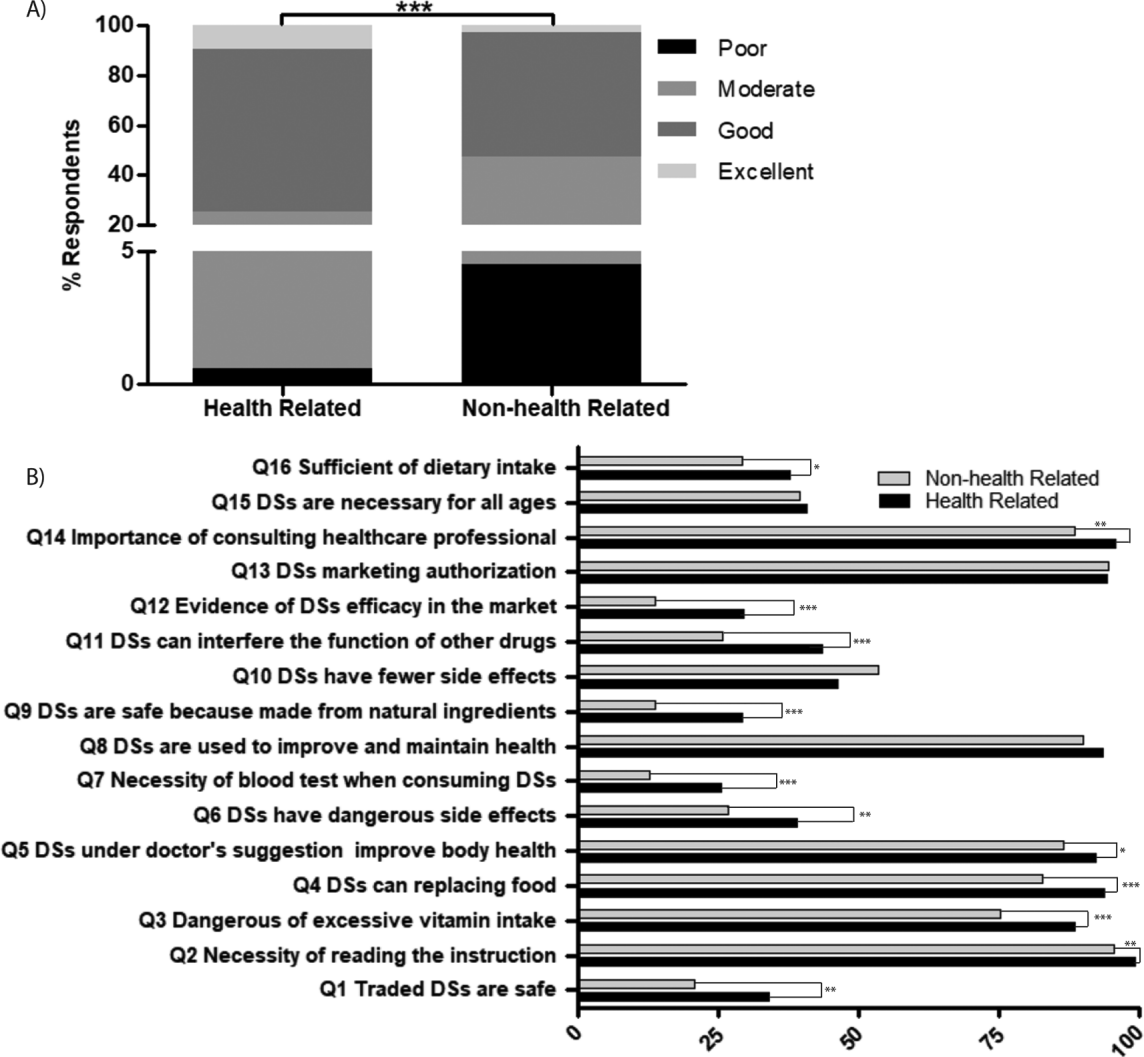
Respondent’s knowledge on DS. (A) comparison of respondents’ level of knowledge based on their background work/study field, (B) comparison of correct answers based on their background work/study field, *** *p-value<0.000*

In a set of questionnaires containing 16 items (S1 Table), respondents’ knowledge on DS was measured. The results showed that 7.21% of respondents had excellent knowledge, 59.52% had good knowledge, 31.24% had fair knowledge and 2.03% had poor knowledge on DS. As expected, based on their field of study or work, the level of knowledge was higher in health-related fields compared with non-health-related fields ( *p=0.000,* Figure 2A). The majority of respondents of both groups knew DS function was not to replace food but to increase nutritional value of their diet and improve health conditions (Q4, Q5 and Q8). Unfortunately, almost two thirds remained unaware that DS were still needed even though they already consumed a healthy diet (Q16), so DS were necessary for all ages (Q15). Moreover, almost all respondents understood that prior to consuming DS, they needed to consult a doctor or pharmacist and read the instructions or the label (Q2 and Q14). The respondents were also aware that before releasing to the market, DS needed a permit from an authorized organization in Indonesia (Q13). However, they were still unaware that not all traded DS were safe to consume (Q1), because some were illegally traded and not all provided evidence to support their beneficial claims (Q12). Regarding side effects, most respondents understood that consuming too many vitamins was harmful (Q3), but they still had a mindset that natural products were safe to consume because they come from nature (Q9). However, their awareness about DS side effects (Q6, Q10) and possible DS-drug interaction (Q11) needed improvement. The Kruskal-Wallis test showed significant differences in the answers between the two groups ( Figure 2B), except for DS function (Q8), side effect DS side effects (Q10), authorization before releasing to the market (Q13) and the necessity of DS for all ages (Q15).

### Determinants of DS consumption practice

To measure predictors of DS consumption of this study, binary logistic regression was performed. For supplement consumption, the univariate analysis of binary logistic regression showed respondents with higher education, working, having confirmed COVID-19 status, married status and have higher self-reported economy status were more likely to consume DS. Using those variables, multivariate analysis of a binary logistic regression model showed significant positive predictors for consuming DS were the health-related field of work or study (AOR: 1.733, 95% CI 1.109-2.709). However, respondents reporting no confirmed COVID-19 status were less likely to consume DS (AOR 0.524, 95% CI 0.291-0.944), and as expected, respondents with a lower self-reported economic status were less likely to consume DS post-Delta wave COVID-19 pandemic (AOR 0.309, 95% CI 0.124-0.769) proving the lack of accessibility due to financial constraints. The model estimated the overall accuracy of 77.8% and explained 9.9% of the variation in supplement consumption for preventing COVID-19 (Omnibus tests of model coefficients chi-square: 36.330, p=0.000). Table 2 showed the predictor factors of DS consumption practice.

**Table 2.** Predictor factors of DS consumption using binary logistic regression.

	Univariate			Multivariate		
Predictor factors	p-value	COR	95% CI	p-value	AOR	95% CI
**Age**						
18-25	0.618	0.718	0.196-2.633			
26-35	0.589	1.477	0.358-6.096			
36-45	0.331	2.104	0.470-9.428			
46-55	0.361	2.045	0.441-9.491			
>55		1				
**Sex**						
Male	0.939	0.984	0.651-1.488			
Female		1				
**Location**						
Main Island	0.495	1.196	0.715-1.999			
Outside Main Island		1				
**Education**						
High School graduate	**0.005**	0.373	0.187-0.747			
Diploma or Undergraduate	0.235	0.647	0.315-1.328			
Post graduate		1				
**Work**						
Employed	**0.000**	2.341	1.454-3.768			
Unemployed		1				
**Field**						
Health-related	0.096	1.418	0.940-2.138	**0.016**	1.733	1.109-2.709
Non-health-related		1				
**Confirmed COVID-19**						
Never	**0.011**	0.475	0.268-0.841	**0.031**	0.524	0.291-0.944
Ever		1				
**Marital status**						
Married	**0.000**	2.484	1.489-4.145			
Unmarried		1				
**Economy status**						
Below average	**0.003**	0.260	0.107-0.629	**0.012**	0.309	0.124-0.769
Average	0.292	0.745	0.431-1.288			
Upper average		1				

Notes: Chi-square for multivariate analysis 1.505, p=0.982, Variables with p-value<0.25 in univariate analysis were subjected to multivariate analysis. Variables with p-value<0.05 in multivariate analysis were subjected as significant predictive factors. AOR: adjusted odds ratio; COR: crude odds ratio; 95% CI: 95% confidence interval

### Predictive factors of DS knowledge

Ordinal logistic regression was performed to investigate the respondents’ knowledge of DS. The results of both univariate and multivariate analyses were consistent in showing that sociodemographic factors such as age, sex, domicile, education, work status, COVID-19 experience, marital status, economic status, and work/study background were not significant predictors of respondents' knowledge regarding DS consumption ( Table 3). Additionally, DS consumption itself was not found to be a significant factor affecting knowledge.

**Table 3.** Predictor factors of DS knowledge using ordinal regression.

	Univariate			Multivariate		
Predictor Factors	p-value	COR	95% CI	p-value	AOR	95% CI
**Age**						
18-25	**0.075**	0.361	0.117-1.110	0.171	0.441	0.137-1.421
26-35	0.347	0.564	0.171-1.858	0.392	0.592	0.178-1.967
36-45	**0.058**	0.310	0.092-1.039	0.061	0.312	0.092-1.056
46-55	**0.119**	0.376	0.110-1.287	0.123	0.376	0.108-1.303
>55		1			1	
**Sex**						
Male	**0.134**	1.303	0.922-1.840	0.240	1.242	0.865-1.782
Female		1			1	
**Location**						
Main Island	0.437	1.189	0.769-1.838			
Outside Main Island		1				
**Education**						
High School graduate	0.348	0.788	0.480-1.296			
Diploma or Undergraduate	0.900	0.968	0.584-1.604			
Post graduate		1				
**Work**						
Employed	**0.223**	1.246	0.875-1.774	0.392	1.261	0.741-2.145
Unemployed		1			1	
**Field**						
Health-related	0.275	0.824	0.581-1.167			
Non-health-related		1				
**Confirmed COVID-19**						
Never	0.445	0.853	0.567-1.284			
Ever		1				
**Marital status**						
Married	0.428	1.160	0.804-1.672			
Unmarried		1				
**Economy status**						
Below Average	0.676	1.189	0.527-2.682			
Average	0.324	1.234	0.812-1.874			
Upper Average		1				
**Supplement consumption**						
No	**0.237**	1.278	0.851-1.921	0.222	1.294	0.856-1.959
Yes		1			1	

Notes: Chi-square for multivariate analysis 66.101, p=0.967, Variables with p-value<0.25 in univariate analysis were subjected to multivariate analysis. Variables with p-value<0.05 in multivariate analysis were subjected as significant predictive factors. AOR: adjusted odds ratio; COR: crude odds ratio; 95% CI: 95% confidence interval.

## Discussion

The COVID-19 pandemic has changed most aspects of human lifestyle including the economy, transportation and of course, the medical field. It has been three years since WHO announced the pandemic, and despite declining cases, the status has not been revoked. Even now, researchers are still actively seeking and revealing new knowledge on COVID-19, including finding preventive measures and definitive drugs for post-COVID complications and long term COVID symptoms. DS as a preventive measure was highly investigated to avoid advanced stages of COVID-19. Several research studies also explored the possibility of DS to reverse the pathophysiology of COVID-19 ( Caballero-García *et al.*, 2021), as well as a complementary therapy for patients with COVID-19 and preventive therapy against lung diseases ( Zhang & Liu, 2020). Several studies including clinical trials, endeavored to prove that DS effectively reduced disease severity, shortened the length of stay and prevented infection by boosting the immune system ( Pinnawala *et al.*, 2021). Unfortunately, those clinical trials failed to prove DS ability to do so ( Amin & Drenos, 2021; Brunvoll *et al.*, 2022; Murai *et al.*, 2021; Thomas *et al.*, 2021). However, increasing DS consumption and sales have been reported worldwide including in the US, Europe and Asia ( d'Arqom *et al.*, 2021; Hamulka *et al.*, 2021; Karlsson *et al.*, 2021; Khabour & Hassanein, 2021; Lordan, 2021) with self-consumption practice, without consultation with healthcare professionals, and still observed in Asia, Africa and America ( d’Arqom et al., 2021, ( Quispe-Cañari *et al.*, 2021; Sadio *et al.*, 2021; Wegbom *et al.*, 2021; Yasmin *et al.*, 2022). Even though the self-consumption of DS is considered safe, their side effects in high-risk populations, especially individuals with several genetic backgrounds, comorbidities and patients on prescribed medication, could be unpleasant. They might experience unwanted effects due to drug and disease interaction ( Agbabiaka *et al.*, 2018; Asher *et al.*, 2017; Ekor, 2014; Peng *et al.*, 2004).

### Practice of dietary supplement consumption and its predictive factors

Our study found increasing DS consumption, as more than three quarters of respondents consumed DS during the Delta wave of the COVID-19 pandemic, with only 59.52% consuming DS before the outbreak. Forty percent of the respondents did not consult with healthcare professionals before consuming DS for more than 3 months. This practice was also found before and during the pandemic, in parallel with other similar studies, such as in 1579 US citizens ( Blendon *et al.*, 2013), 105 athletes in Saudi Arabia ( Aljaloud & Ibrahim, 2013), 651 students in Saudi Arabia ( Almegewly *et al.*, 2022) and 48,925 Japanese adults ( Chiba & Tanemura, 2022). This consumption was significantly associated with middle age, higher education, higher economic status, marital status, receiving a diagnosis of COVID-19 and employment status. However, our regression model showed that only health-related field of work or study was a positive predictive factor, while never confirmed COVID-19 status and lower self-reported economic status were negative predictors.

Younger respondents might feel healthier and at lower risk of obtaining severe stage of COVID-19 ( Libertini *et al.*, 2019; Perrotta *et al.*, 2020), while older respondents might be unaware of the risk and the disease, despite being more prone to transmission ( Wolf *et al.*, 2020). Another possible reason was they might be unaware of the risk of interaction between DS and their diseases or drugs consumed ( Agbabiaka *et al.*, 2018; Alkhalidi *et al.*, 2019). However, a high possibility existed that respondents having received a diagnosis of COVID-19 would like to prevent re-infection by consuming DS ( Ali, 2020; Shahbaz *et al.*, 2022). Married and working respondents might consume DS due to their responsibilities to avoid infection and need to fulfil their duties to their family members. This finding was similar to the study in Indonesia during the first year of the pandemic ( d'Arqom *et al.*, 2021), Saudi Arabia ( Radwan *et al.*, 2022) and Lebanon ( Mohsen *et al.*, 2021). Higher economic status respondents were able to buy the DS and might have a higher responsibility in their workplace; thus, they had consumed DS. Similar phenomena were also reported in Indonesia and Saudi Arabia ( d'Arqom *et al.*, 2021; Radwan *et al.*, 2022). A study on 11,240 US adults from 2011 to 2014 also reported similar finding, as consumption of DS was higher among those earning higher incomes ( Cowan *et al.*, 2018).

Among the two groups, respondents with non-health-related background were more likely to consume DS for more than three months compared with respondents with health-related backgrounds. Despite of not being in the medical and healthcare career line, non-health background respondents’ have better long-term compliance. According to Biesalski and team, long term compliance in DS such as multivitamin and mineral supplement brings benefit to general health from developing lifestyle disease and was also documented safe in over 10 years of consumption in the clinical trial ( Biesalski & Tinz, 2017). However, this miraculous finding does not apply to all DSs. Stranges *et al.* (2007) found that prolonged use of selenium, an ingredient commonly found in mainstream ingestible beauty supplements, can lead to an increased risk of developing type 2 diabetes later in life. Therefore, there are potential detrimental effects of long-term compliance with such supplements ( Stranges *et al.*, 2007).

DS consumption practice was also associated with the method in acquiring DS. Drug stores were the primary vendor to purchase DS, followed by e-commerce platform and any store selling DS. This finding supported the significant presence of online shopping, due to its convenience and ease during the COVID-19 pandemic ( Eger *et al.*, 2021; Gu *et al.*, 2021). Moreover, respondents with health-related background were more likely to buy using e-commerce platforms than the non-health-related respondents. A study involving 34,488 Italians did not investigate the work/study background of the respondents as one of the predictive factors for online shopping; however, they found that younger age, higher education, female, good economic status and extended working hours comprised positive predictors for online shopping ( Dominici *et al.*, 2021). This might be one of the reasons, because healthcare professionals and medical students have long working hours ( Dyrbye *et al.*, 2017; Shreffler *et al.*, 2020), including during the COVID-19 pandemic ( Razu *et al.*, 2021). However, this finding raises concerns about the regulatory control of e-commerce platforms and physical stores selling dietary supplements. Unlike drug stores, where pharmacists are available to offer advice and address concerns about supplements and their potential side effects, e-commerce platforms and other stores selling DS may not provide access to experts.

### Knowledge on dietary supplements and its predictive factors

Moreover, our study found that one half of respondents possessed good knowledge levels about DS, with only 2% having poor knowledge. However, our findings might be biased because almost two thirds of our respondents had health-related work/study backgrounds. However, the comparison of DS knowledge in both groups showed respondents with non-health-related backgrounds possessed less knowledge about DS, supporting our above-mentioned concern on the purchasing habits out of drug store. This finding differed from that of 351 students in Saudi Arabia, reporting no difference between the knowledge level of health science students with those of non-health science students ( Alowais & Selim, 2019). Unfortunately, most were still partially unaware of the safety and side effects of DS on the market and the necessity of DS consumption. This hurdle was also found among 537 US healthcare professionals ( Kemper *et al.*, 2003) and 179 US students pharmacist ( Axon *et al.*, 2017). Our study found that their level of knowledge was not determined by sociodemographic factors and DS consumption in the post-delta wave of the COVID-19 pandemic, as no significant predictor factors were found in univariate and multivariate analysis.

Even though sociodemographic factors did not affect the respondents’ knowledge of DS, we need to be aware about health inequality that has been highlighted during the COVID-19 pandemic and has been discussed extensively ( McGrail *et al.*, 2022; Mishra *et al.*, 2021). In Indonesia itself, in 2017, the WHO and Ministry of Health Republic of Indonesia reported an existing gap on health status in Indonesia related to economic status, education level, occupation, employment status, age, sex, place of residence and subnational region. These disparities include low consumption of fruits and vegetables, known as the main source of vitamins and minerals from food ( WHO & Kemenkes, 2017). Several other studies also reported these health disparities ( Haemmerli *et al.*, 2021; Mulyanto *et al.*, 2019). Reportedly, the implementation of national coverage insurance has resulted in a decrease in health disparities attributed to differences in economic status ( Warsito & Adisasmito, 2020). Regrettably, the COVID-19 pandemic has led to an increase in socio-economic disparity in urban areas, while rural areas with the highest COVID-19 cases have experienced a decrease, yet the impact of this trend on health inequalities remains unreported ( Brata *et al.*, 2021).

### Limitation and recommendation

Even though the internet-based survey was conducted conveniently and efficiently, the questionnaire might not have reached the respondents in remote areas of Indonesia due to limited internet access. Moreover, limited interaction with the respondents might have created a biased responses to the questionnaire. Furthermore, the sampling methods did not represent the distribution of Indonesian adults. Thus, more respondents and better questionnaire outreach are needed to draw a complete picture of the practices and knowledge level of DS consumption post-delta wave of the COVID-19 pandemic. Despite the limitations, this study brings an important understanding on the practice and knowledge of DSs consumption in Indonesia, which have reconfigured our food and dietary psychology and habits due to the COVID-19 pandemic.

This study showed the practices and knowledge level of DS consumption in Indonesia post-delta wave of the COVID-19 pandemic. Even though the self-consumption of DS is considered safe, its side effects in high-risk populations, namely individuals with several comorbidities and patients on prescribed medication, might experience unwanted effects due to drug and disease interaction. Therefore, collaborative efforts from a multitude of organisations including medical doctors, pharmacists and governmental bodies to uphold their responsibilities to educate and provide essential and easily understandable information available to the general public. Based on this current study, the practice of self-consumption of DS in Indonesia is increasing, and this pattern is expected to increase further in the coming years. Hence, knowledge of DS is necessary to avoid detrimental effects that might occur in the future. This could be done by increasing accessibility of information with better labelling and educating youth, the future consumers to empower users to make wise choices on DS.

## Data Availability

Mendeley Data: Practice and Knowledge DS Indonesian Adult. doi: 10.17632/n2fdtbwrxb.2 ( d'Arqom, 2022) This project contains the following underlying data:
- Data 514 analysis.xlsx - Erratum Data 514 analysis.xlsx (correction of coding variables, all started from 0) This project contains the following extended data:
- Supplementary file.docx (contains the survey instrument and differences between DS consumption practice of respondents with health-related background and non-health-related background) Data are available under the terms of the Creative Commons Attribution 4.0 International license (CC-BY 4.0).
