# Supplementary material for: Practice and knowledge of dietary supplement consumption among Indonesian adults post-delta wave of the COVID-19 pandemic
Source: F1000Res. 2023 May 4;12:3. Originally published 2023 Jan 3. [Version 2] doi: 10.12688/f1000research.129045.2 (PMC10352623; doi:10.12688/f1000research.129045.2)
Supplement: Peer review history

# Peer review: Reviewer response for version 2

**DOI:** 10.5256/f1000research.147596.r182345

**Published:** 2023-07-27

**Authors:** Bamgboye M. Afolabi

**Affiliations:** 1 Nigerian Institute of Medical Research, Lagos, Nigeria

**Brief summary:**

 The reviewed paper, titled “Practice and knowledge of dietary supplement (DS) consumption among Indonesian adults post-delta wave of the COVID-19 pandemic” was an internet-based survey which focused primarily on how dietary supplements was consumed by a statistically determined group of people in Indonesia, during the COVID-19 pandemic, as well as during the post-Delta wave period. The authors utilized descriptive method of analysis to illustrate the consumption pattern (frequency, duration, aim, preferrable dosage form), familiarity with and side-consequences of DS among the respondents.

 This is a well written paper which throws more light on the consumption of DS not only in Indonesia but throughout the world. Other reviewers have commented extensively on the statistical analysis and inferences in this paper. Authors should go over the syntaxes and tenses as I have suggested in the review paper which I am submitting with comments and highlighted sentences or words, LINK.

General Comments:

**Introduction: **

- The subject matter of this manuscript, consumption of DH, is of global concern and the period of study was a time of worldwide pandemic. Currents of vital information on these two issues are vital to the scientific world. The introduction segment of the paper, consisting of four paragraphs, is well written but few sentences therein require correction of typographical errors and tenses.
- Furthermore, the authors should probably have presented DS consumption from a wider area of the world so that readers could have a better idea of the depth of the health problems that the study was trying to unravel.

**Method:**

- This segment of the manuscript was divided into five segments – Study design, Participants, Data collection, Ethical consideration, and Analytical procedures. Here, the authors should probably have given a brief description of the study site, including the population of Indonesia and some geographical information, if the sample size was adequate for statistical inference, though this really depends on the response rate as this was an on-line study.
- One of the minor drawbacks of this section is the syntax of the last sentence in the data collection section which has been referred to and corrected in the review.

**Result:**

- The Result segment of the reviewed paper contains most of the sentences or words that need correction. First, there is no need to express percentages beyond one decimal place. In fact, figures are best expressed as whole numbers. This has been reflected in the reviewed paper.
- Secondly, some of the sentences in this segment of the paper need to be rephrased to make sense to the readers. One minor observation was that the first column in Table 1 may be re-arranged, putting Marital status and Economic status after Sex followed by Education, Work, Field of work/study and lastly Confirmed COVID-19.
- Further, Figure 1 is too compact and should probably be spread out and given a relatively clearer explanation, especially Figures 1 C and D. Figure 1 A refers to co-morbidities of the respondents, but this should be explained further. Is it that these disease entities occur together in the same patient (co-morbidity) or they exist differently, in different prevalence, among the study participants? This should be made clear to readers. The following questions in the questionnaire needs further clarification – Q6: DSs have dangerous side effects and Q10: DS have fewer side effects. The reason is that Q6 does not preclude Q10. DSs fewer but dangerous side effects and fewer or more DSs can have dangerous side effects. 

**Discussion:**

- It would be good to include references from different regions of the world, including Africa, which was also ravaged by the COVID-19 pandemic and where DSs are also consumed in large quantities before, during and after the pandemic.
- The authors quoted Biesalski  & Tinz, 2017 as having a “miraculous” finding but this word – miraculous – should be deleted from this manuscript because it is subjective, and others may not find it miraculous. Some statements in this segment of the manuscript are incomplete and authors have been advised to complete them in the reviewed paper.

**Limitation and recommendations:**

- A statement in the second paragraph in this segment, also highlighted in yellow as others that need correction, has been reconstructed. The authors should take a look at it and possibly effect the recommended changes.

Overall, this is a well-written paper with good ideology. I would like to congratulate the authors for their efforts in producing a splendid work. The authors may wish to go over the reviewed paper and make possible changes to the areas that need correction.

 Congratulations again.

Is the work clearly and accurately presented and does it cite the current literature?

Yes

If applicable, is the statistical analysis and its interpretation appropriate?

I cannot comment. A qualified statistician is required.

Are all the source data underlying the results available to ensure full reproducibility?

Yes

Is the study design appropriate and is the work technically sound?

Yes

Are the conclusions drawn adequately supported by the results?

Yes

Are sufficient details of methods and analysis provided to allow replication by others?

Yes

Reviewer Expertise:

Tropical Diseases, Women's Health, Adolescent Nutrition.

I confirm that I have read this submission and believe that I have an appropriate level of expertise to confirm that it is of an acceptable scientific standard.

# Peer review: Reviewer response for version 2

**DOI:** 10.5256/f1000research.147596.r182347

**Published:** 2023-07-17

**Authors:** Siti Maisharah Sheikh Ghadzi

**Affiliations:** 1 School of Pharmaceutical Sciences, Universiti Sains Malaysia, Minden Heights, Pulau Pinang, Malaysia

**Brief summary**

 This manuscript was designed with the aim to investigate the practice and knowledge of the general public in Indonesia regarding DS during the post-Delta wave of the COVID-19 pandemic. In addition, the predictive factors of DS consumption and level of knowledge were further analyzed.

**General comments**

**Method**

- Questionnaire: more information is needed, especially on the validity tests. What type of validity test was conducted?
- How do the authors determine the eligibility of the pharmacologist, pharmacist and psychologist to validate the questionnaire? Are 4 reviewers sufficient? Please explain with references.
- How do the authors determine whether the number of subjects for the pilot study is sufficient? Please add the reference as well.
- How did the authors determine the level of knowledge based on the score? Were any standard classifications used, (example: Bloom’s cut off point)? Please add the explanation and reference.

**Results**

- Figure 2b describes the results from Kruskal Wallis. But the information on the use of Kruskal Wallis is not described in the method section. Were the data not normally distributed? Please revise accordingly.
- Table 2 and Table 3. It is not clear on why the demographic data was not included in the logistic regression analysis. Please describe the reason, and revise if needed. Perhaps because it was not significant in the univariate analysis? If yes, then my suggestion is to describe in the statistical analysis, in which to add that the significance variables in the univariate analysis is further investigated using univariate and multivariable logistic regression. Please revise accordingly.
- Table 2: why are Education, Work and Marital statuses not included into the multivariable analysis even though it is significant in the univariate regression? Why is Field included in the multivariable logistic regression even though it is not significant in the univariate regression?

**Discussion**

- Paragraph 1: It would be good to include the examples of side effects in high risk population, and unwanted effects dur to drug and disease interaction.
- Paragraph 2: any potential reason for the results of regression model? It would be good to add the explanation on the potential reason.

Is the work clearly and accurately presented and does it cite the current literature?

Yes

If applicable, is the statistical analysis and its interpretation appropriate?

Partly

Are all the source data underlying the results available to ensure full reproducibility?

Yes

Is the study design appropriate and is the work technically sound?

Partly

Are the conclusions drawn adequately supported by the results?

Yes

Are sufficient details of methods and analysis provided to allow replication by others?

Yes

Reviewer Expertise:

Clinical pharmacy, endocrine, pharmacokinetic/pharmacodynamic modelling, pharmacotherapy

I confirm that I have read this submission and believe that I have an appropriate level of expertise to confirm that it is of an acceptable scientific standard.

# Peer review: Reviewer response for version 2

**DOI:** 10.5256/f1000research.147596.r171989

**Published:** 2023-05-19

**Authors:** Mikołaj Kamiński

**Affiliations:** 1 Department of Treatment of Obesity, Metabolic Disorders and Clinical Dietetics, Poznań University of Medical Sciences, Poznań, Poland

Thank you for the responses. All my comments were responded.

Is the work clearly and accurately presented and does it cite the current literature?

No

If applicable, is the statistical analysis and its interpretation appropriate?

Partly

Are all the source data underlying the results available to ensure full reproducibility?

Partly

Is the study design appropriate and is the work technically sound?

No

Are the conclusions drawn adequately supported by the results?

Partly

Are sufficient details of methods and analysis provided to allow replication by others?

Partly

Reviewer Expertise:

Public Health

I confirm that I have read this submission and believe that I have an appropriate level of expertise to confirm that it is of an acceptable scientific standard.

# Peer review: Reviewer response for version 1

**DOI:** 10.5256/f1000research.141700.r168163

**Published:** 2023-03-30

**Authors:** Mikołaj Kamiński

**Affiliations:** 1 Department of Treatment of Obesity, Metabolic Disorders and Clinical Dietetics, Poznań University of Medical Sciences, Poznań, Poland

The manuscript presents a study focusing on the practices and knowledge level of dietary supplement (DS) consumption in Indonesia, particularly after the COVID-19 pandemic. The authors have provided a comprehensive background on the topic and presented their findings based on a well-structured questionnaire. However, there are certain aspects that need to be addressed to improve the manuscript's overall quality and clarity.

**Major Comments:**

1. The authors mention that the study was conducted after the COVID-19 pandemic. However, considering that the pandemic is still ongoing and that the WHO has not revoked the pandemic status, it would be better to rephrase the context of the study as "during the COVID-19 pandemic" or "in the post-delta wave of the COVID-19 pandemic" instead of "after the COVID-19 pandemic."
2. The manuscript would benefit from a clear description of the study design, including the sampling method, sample size, and the study period. It is essential to provide these details to assess the study's robustness and reliability.
3. The authors should consider providing a demographic breakdown of the respondents, including age, gender, geographic distribution, and other relevant characteristics. This information is crucial to understand the generalizability of the study findings.
4. The authors should provide more details about the questionnaire, including how it was developed, its validation process, and how the knowledge levels of respondents were assessed.
5. The authors have highlighted several limitations of the study, such as the sampling method, questionnaire outreach, and potential bias in the responses. They should consider discussing potential strategies to address these limitations in future studies to provide a more comprehensive understanding of DS consumption practices and knowledge levels in Indonesia.
6. The discussion section could be better organized by separating the key findings and relating them to the existing literature. It is essential to highlight the novel aspects of the study and the implications of the findings for policy and practice.
7. Given the study's focus on practices and knowledge levels, it would be helpful to include recommendations for public health interventions to promote safe and informed DS consumption, such as educational campaigns or regulatory measures.

**Minor Comments:**

1. In several instances throughout the manuscript, the authors use the term "DS" without defining it first. It would be helpful to define the term "dietary supplements" before using the abbreviation "DS" to improve readability.
2. Some sentences are lengthy and could be broken down into shorter, more concise statements for better readability.
3. The manuscript would benefit from a thorough proofreading to correct grammatical errors, inconsistencies in citations, and formatting issues.

In conclusion, the manuscript presents an interesting study examining the practices and knowledge level of dietary supplement consumption in Indonesia during the COVID-19 pandemic. However, several aspects need to be addressed to improve the manuscript's quality and clarity. By addressing these concerns, the authors can present a more robust and reliable study that contributes to the understanding of dietary supplement consumption in the context of the ongoing pandemic.

Is the work clearly and accurately presented and does it cite the current literature?

No

If applicable, is the statistical analysis and its interpretation appropriate?

Partly

Are all the source data underlying the results available to ensure full reproducibility?

Partly

Is the study design appropriate and is the work technically sound?

No

Are the conclusions drawn adequately supported by the results?

Partly

Are sufficient details of methods and analysis provided to allow replication by others?

Partly

Reviewer Expertise:

Public Health

I confirm that I have read this submission and believe that I have an appropriate level of expertise to state that I do not consider it to be of an acceptable scientific standard, for reasons outlined above.

# Response

**Published:** 2023-04-28

**Authors:** Annette d'Arqom

**Affiliations:** Airlangga University, Indonesia

**Response to Review and Editor Comments**

**(Manuscript Number: **ID 129045 **)**

 We are very much thankful to the reviewers for their deep review. I have revised the manuscript in the light of their useful suggestions and comments. We hope the revision improves the paper to a level of their satisfaction. Number wise answers to the specific suggestions/comments are as follows:

**Response to Reviewer: 1**

**Comments to the Author**

 The manuscript presents a study focusing on the practices and knowledge level of dietary supplement (DS) consumption in Indonesia, particularly after the COVID-19 pandemic. The authors have provided a comprehensive background on the topic and presented their findings based on a well-structured questionnaire. However, there are certain aspects that need to be addressed to improve the manuscript's overall quality and clarity.

**Major Comments:**

 1. The authors mention that the study was conducted after the COVID-19 pandemic. However, considering that the pandemic is still ongoing and that the WHO has not revoked the pandemic status, it would be better to rephrase the context of the study as "during the COVID-19 pandemic" or "in the post-delta wave of the COVID-19 pandemic" instead of "after the COVID-19 pandemic."

*Response: Thank you very much for bringing up this matter. We change the sentences into post-delta wave of the COVID-19 pandemic as in the line 350 and 354.*

 2. The manuscript would benefit from a clear description of the study design, including the sampling method, sample size, and the study period. It is essential to provide these details to assess the study's robustness and reliability.

*Response: Thank you very much for pointing out this issue. We did mention the sampling methods and the study period in the line 52-55 (“The questionnaire was distributed to the adult population in Indonesia through email and social media using convenience sampling methods from October to December 2021, and multiple responds from single device were prevented based on respondents’ IP address.”); and the sample size in the line 62-65 (“Sample size was calculated using sample size calculator ( www.raosoft.com) with 5% margin of error, 95% confidence level, and population number filled with 100,000, resulting in 383 minimum respondents.”).*

****

 3. The authors should consider providing a demographic breakdown of the respondents, including age, gender, geographic distribution, and other relevant characteristics. This information is crucial to understand the generalizability of the study findings.

*Response: Thank you very much for your suggestion. The sociodemographic characteristic of the respondents was provided in table 1.*

 4. The authors should provide more details about the questionnaire, including how it was developed, its validation process, and how the knowledge levels of respondents were assessed.

*Response: Thank you very much for bringing up this matter. We did mention about the development of the questionnaire and the validation process as in the line 78-88 (“The questionnaire was modified from the Prevalence and Awareness Concerning Dietary Supplement Use among Saudi Adolescents ( Alfawaz et al., 2020) and Knowledge about dietary supplements and trust in advertising them: Development and validation of the questionnaires and preliminary results of the association between the constructs ( Karbownik et al., 2019). The questionnaire was combined and validated by two pharmacologists, a pharmacist, and a psychologist, and was further tested on 20 respondents to ensure that the content and terms used in the questionnaire were relevant and understandable. The reliability measurement on the survey data showed a Cronbach alpha coefficient of 0.750 for DS knowledge. As the reliability value for the preliminary pilot testing is at an acceptable level, the real data collection for this study was proceed.”). Moreover, the assesment the knowledge level of the respondents was mention in the line 102-105 (“The answers to the knowledge survey were measured using correct/wrong/does not know options measured as 1/0/0. The total score was the summation of the 16 question scores and was grouped in four categories based on the total score of each respondent: poor (0-4), moderate (5-8), good (9-12) and excellent (13-16).”)*

 5. ****The authors have highlighted several limitations of the study, such as the sampling method, questionnaire outreach, and potential bias in the responses. They should consider discussing potential strategies to address these limitations in future studies to provide a more comprehensive understanding of DS consumption practices and knowledge levels in Indonesia.

*Response: Thank you very much for pointing out this matter. We did provide the strategies to address this limitation for better future studies as in the line 348-350 (“Thus, more respondents and better questionnaire outreach are needed to draw a complete picture of the practices and knowledge level of DS consumption post-delta wave.”)*

****

 6. The discussion section could be better organized by separating the key findings and relating them to the existing literature. It is essential to highlight the novel aspects of the study and the implications of the findings for policy and practice.

*Response: Thank you very much for your suggestion. We divide the discussion part into three sections: (1) practice of dietary supplement consumption and its predictive factors, (2) knowledge on dietary supplements and its predictive factors, and (3) limitation and recommendation.*

****

 7. Given the study's focus on practices and knowledge levels, it would be helpful to include recommendations for public health interventions to promote safe and informed DS consumption, such as educational campaigns or regulatory measures.

*Response: Thank you very much for bringing up this matter. We did include the recommendation for promoting the safe and informed DS consumption as in the line 358-361 (“Therefore, collaborative efforts from a multitude of organisations including medical doctors, pharmacists and governmental bodies to uphold their responsibilities to educate and provide essential and easily understandable information available to the general public.”) and 364-365 (“This could be done by increasing accessibility of information with better labelling and educating youth, the future consumers to empower users to make wise choices on DS.”)*

**Minor Comments:**

 8. ****In several instances throughout the manuscript, the authors use the term "DS" without defining it first. It would be helpful to define the term "dietary supplements" before using the abbreviation "DS" to improve readability.

*Response: Thank you very much for pointing out this issue. We did define dietary supplements as DS in the first sentence of the abstract.*

****

 9. Some sentences are lengthy and could be broken down into shorter, more concise statements for better readability.

*Responses: Thank you very much for the suggestion. We re-write some sentences to increase the readability, for example in the line 19-26, 31-36, 222-226, 290-293, 307-311, and 336-342.*

****

 10. The manuscript would benefit from thorough proofreading to correct grammatical errors, inconsistencies in citations, and formatting issues.

*Responses: Thank you very much for the suggestion. Our manuscript has been reviewed by a English native speaker. We do hope it meets your expectation.*

 11. In conclusion, the manuscript presents an interesting study examining the practices and knowledge level of dietary supplement consumption in Indonesia during the COVID-19 pandemic. However, several aspects need to be addressed to improve the manuscript's quality and clarity. By addressing these concerns, the authors can present a more robust and reliable study that contributes to the understanding of dietary supplement consumption in the context of the ongoing pandemic.

*Responses: Thank you very much for your review. We do hope that our revision does improve the *quality and clarity *of the manuscript.*
